# Gut microbiome is associated with radiotherapy response in lung cancer patients with brain metastases

**DOI:** 10.3389/fcimb.2025.1562831

**Published:** 2025-03-10

**Authors:** Fei Liang, Yichu Sun, Jing Yang, Ziqiang Shen, Guangfeng Wang, Jiangrui Zhu, Chong Zhou, Youyou Xia

**Affiliations:** ^1^ Department of Radiation Oncology, the First People's Hospital of Lianyungang/ Lianyungang Clinical College of Nanjing Medical University, Lianyungang, Jiangsu, China; ^2^ Department of Radiation Oncology, The Affiliated Lianyungang Hospital of Xuzhou Medical University/ The First People's Hospital of Lianyungang, Lianyungang, Jiangsu, China; ^3^ Department of Radiation Oncology, Xuzhou Central Hospital, Xuzhou, Jiangsu, China

**Keywords:** lung cancer, brain metastasis, gut microbiome, radiotherapy, efficacy prediction model

## Abstract

**Purpose:**

To investigate the gut microbiome of lung cancer patients with brain metastases undergoing radiotherapy, identify key microorganisms associated with radiotherapy response, and evaluate their potential as biomarkers.

**Methods and materials:**

This study enrolled 55 newly diagnosed lung cancer patients with brain metastases. Fecal samples were collected before radiotherapy and analyzed by 16S rRNA sequencing to assess the gut microbiome’s composition and function. Patients were categorized into response (n=28) and non-response (n=27) groups based on treatment efficacy, and α-diversity, β-diversity, and functional pathways were compared between them. Linear Discriminant Analysis Effect Size was used to identify microbial features associated with treatment efficacy. Logistic regression analyses were performed to evaluate the predictive capacity of clinical and microbial factors for treatment outcomes.

**Results:**

No significant difference in α-diversity was observed between the groups (P > 0.05), but β-diversity differed significantly (P = 0.036). Twelve characteristic microorganisms were identified in the response group, including *g_ Oscillibacter* and *g_ Blautia*, and nine in the non-response group, such as *f_ Desulfovibrionaceae* and *g_ Megamonas*. Metabolic pathways associated with treatment response included ketone body metabolism and pathways related to amyotrophic lateral sclerosis. Multivariate analysis identified *g_Flavonifractor* (odds ratio [OR] = 6.680, P = 0.004), *g_Negativibacillus* (OR = 3.862, P = 0.014), C-reactive protein (OR = 1.054, P = 0.017), and systemic inflammation response index (OR = 1.367, P = 0.043) as independent predictors of radiotherapy response. The nomogram and microbiome models achieved area under the curve (AUC) values of 0.935 and 0.866, respectively, demonstrating excellent predictive performance. Decision curve analysis further confirmed these models provided significant net benefits across risk thresholds.

**Conclusions:**

The composition and functional characteristics of the gut microbiome in lung cancer patients with brain metastases prior to radiotherapy are associated with therapeutic response and possess potential as predictive biomarkers. Further studies are warranted to validate these findings.

## Introduction

Lung cancer (LC) remains the leading cause of cancer-related mortality worldwide, with approximately 10-36% of patients developing brain metastases (BM) during the disease course (Barnholtz-Sloan et al., 2004; Bray et al., 2024). Patients with BM generally face a poor prognosis, characterized by a median survival of 4–10 months and a 5-year survival rate below 5% (Cagney et al., 2017). Radiotherapy (RT) is the primary treatment modality for brain metastases in lung cancer (LC-BM) patients, effectively controlling known brain metastatic lesions and eradicating undetected micrometastases (Weller et al., 2024). However, in real-world clinical practice, some patients experience significant tumor reduction and symptom improvement following RT, while others show limited response or even develop radioresistance and further tumor progression. Although previous studies have identified various clinical and biological factors influencing RT efficacy, including tumor hypoxia, metabolic alterations, immune microenvironment, host immune responses, and overall health status (Youssef et al., 2024), these factors are insufficient to fully explain the heterogeneity in RT outcomes. Many potential factors remain undiscovered and warrant further investigation.

The gut microbiome is a vital regulator of host health, maintaining metabolic balance, immune modulation, and barrier functions, and also directly or indirectly modulating tumor responses to treatment by influencing drug metabolism, transport, enzymatic degradation, and immune reactions (Chrysostomou et al., 2023). For example, in chemotherapy, *Gammaproteobacteria* degrade gemcitabine into its inactive form through cytidine deaminase, thereby diminishing its efficacy (Geller et al., 2017). *Fusobacterium nucleatum* mediates resistance to 5-fluorouracil and oxaliplatin in colorectal cancer by regulating autophagy mechanisms and immune responses (Yu et al., 2017). In contrast, *Bacteroides fragilis* and *Bacteroides thetaiotaomicron* enhance the sensitivity of pancreatic cancer to the FOLFIRINOX (a regimen consisting of fluorouracil, leucovorin, irinotecan, and oxaliplatin) chemotherapy regimen through similar mechanisms (Tintelnot et al., 2023). In immunotherapy, *Bifidobacterium*, *Akkermansia muciniphila*, and *Lactobacillus rhamnosus* GG promote T cell recruitment to tumor sites by modulating antigen-presenting cell (APC) functions, particularly dendritic cells (DCs). This modulation is primarily mediated through the secretion of cytokines such as type I interferon (IFN) and interleukin-12 (IL-12) (Sivan et al., 2015; Routy et al., 2018; Si et al., 2021). For instance, oral administration of live *Lactobacillus rhamnosus* GG induces IFN-β production in DCs via the cGAS/STING pathway, which enhances CD8^+^ T cell cross-priming. Similarly, *Akkermansia muciniphila* restores PD-1 blockade efficacy by recruiting CCR9+CXCR3+CD4^+^ T lymphocytes into the tumor microenvironment in an IL-12-dependent manner. Collectively, these microbiota-driven cytokine modulations and APC activation synergistically enhance the antitumor effects of PD-1/PD-L1 inhibitors. While substantial evidence exists on the interactions between the gut microbiome and chemotherapy or immunotherapy (Li et al., 2024; Li et al., 2024), the influence of the gut microbiome on radiotherapy efficacy remains exploratory and not fully understood or confirmed (Lu et al., 2024). Preclinical studies preliminarily suggest that the gut microbiome can enhance local RT effects and mediate the abscopal effect (a phenomenon where localized treatment induces distant tumor regression) of RT by remodeling the tumor immune microenvironment (Uribe-Herranz et al., 2020). Additionally, the overgrowth of commensal fungi may significantly contribute to radioresistance (Shiao et al., 2021). Clinical studies suggest that gut microbiome composition is closely linked to RT outcomes in solid tumors and may act as potential biomarkers (Yi et al., 2021). For instance, a study identified microbial signatures, including the *NK4A136 and UCG-003 groups* as well as *Eubacterium hallii*, in patients with non-small-cell lung cancer who were treated with concurrent chemoradiotherapy. These features predicted whether patients had progression-free survival beyond 11 months, demonstrating the potential of gut microbiome composition as a predictive biomarker of cancer RT outcomes (Qiu et al., 2023).

**Figure 1:**
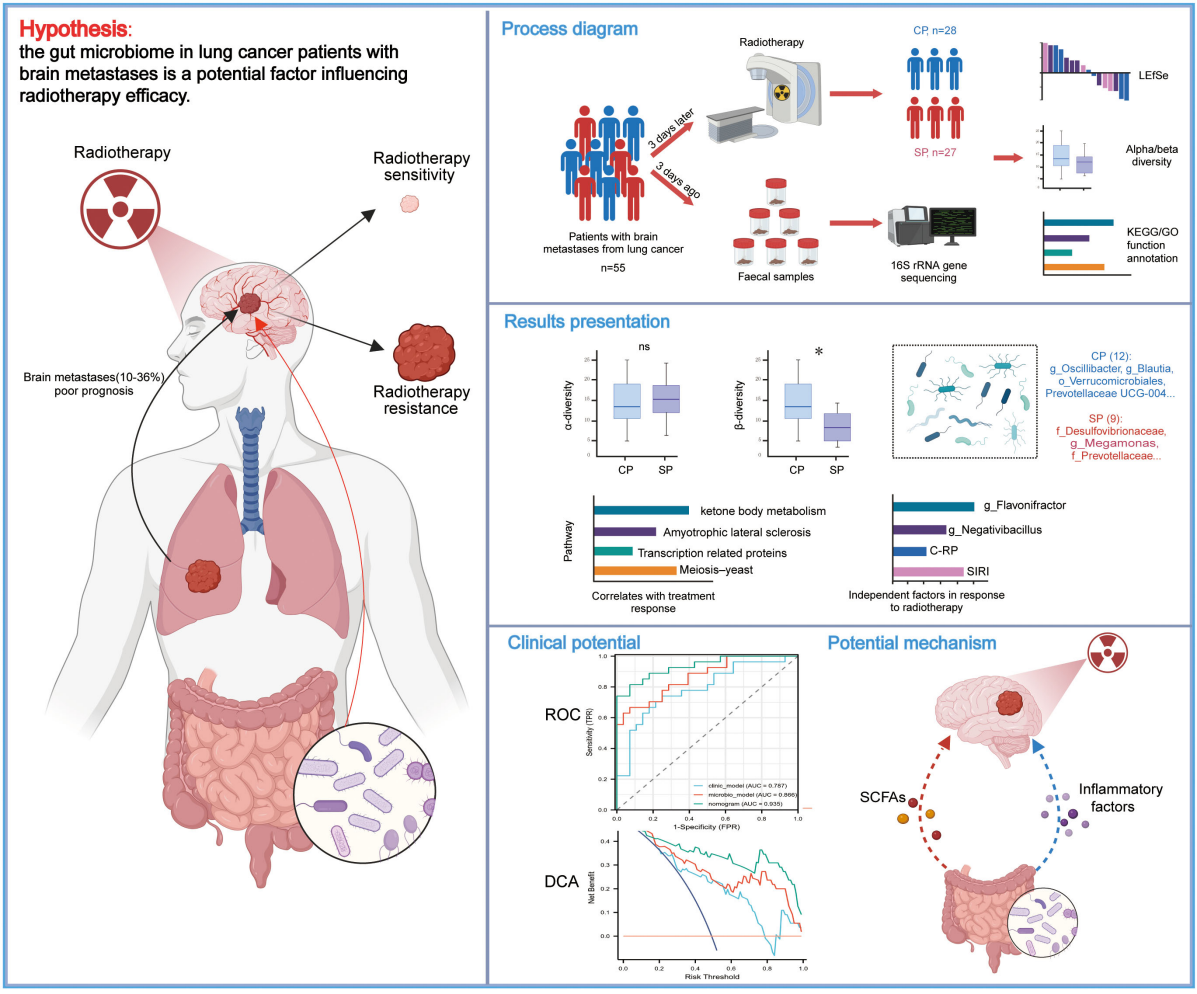
Study design, workflow, and key findings. The schematic diagram illustrates the recruitment of lung cancer patients with brain metastases, collection of fecal samples, 16S rRNA gene sequencing, and subsequent bioinformatics workflows alongside partial statistical analysis results. The figure highlights key microbial and clinical predictors of radiotherapy response, including *g_Flavonifractor*, *g_Negativibacillus*, C-RP, and SIRI, as well as predictive models. Additionally, potential mechanisms by which the gut microbiome may influence radiotherapy response are proposed. CP group, radiotherapy responders; SP group, radiotherapy non-responders; LEfSe, Linear discriminant analysis effect size; C-RP, c-reactive protein; SIRI, systemic inflammation response index; ROC, receiver operating characteristic; DCA, decision curve analysis.

In summary, while radiotherapy is a critical treatment modality for LC-BM, its efficacy exhibits considerable inter-individual variability. Given the gut microbiome’s key regulatory role in various antitumor therapies, we hypothesize that it may influence the therapeutic outcomes of radiotherapy in LC-BM patients and hold potential as a biomarker. To validate this hypothesis, we systematically assessed the gut microbiome’s composition and functionality in LC-BM patients, comparing those with RT responses and those without. This study aims to identify microbial factors that influence RT efficacy and discover biomarkers that predict patient responses. Additionally, it seeks to provide scientific evidence for interventions aimed at enhancing RT outcomes through modulation of the gut microbiome. To provide readers with a comprehensive overview of our study design, objectives, and key findings, we have included a schematic diagram at the end of this section (**Figure 1**).

## Materials and methods

### Participant recruitment and selection

This prospective observational study received approval from the Ethics Committee of the First People’s Hospital of Lianyungang City (KY-20230505001-01), and all participants gave written informed consent. Between May 2023 and August 2024, sixty-two patients with newly diagnosed BM from lung cancer were enrolled, all of whom received RT for BM. The inclusion criteria included: (1) an initial LC-BM diagnosis confirmed by histopathology or typical magnetic resonance imaging (MRI) findings; (2) a minimum of 3 weeks since the last systemic therapy; (3) capability to provide complete fecal samples and consent to participate in follow-up assessments. Participants were excluded if they had received antibiotics, probiotics, or steroids within four weeks prior to RT; had a history of gastrointestinal diseases or digestive tract surgery; or had severe cardiovascular, metabolic, neurological diseases, or other comorbidities making study participation unsuitable.

### Radiotherapy protocol formulation and clinical data collection

Radiotherapy plans for BM were formulated by the expert team at our institution’s RT Center, based on patients’ medical histories, imaging data, and multidisciplinary consultation results, then implemented using the Varian RT system. All patients received either whole-brain radiotherapy (WBRT) alone, WBRT combined with simultaneous integrated boost (SIB), or fractionated stereotactic radiotherapy (FSRT), with RT doses following the NCCN Clinical Practice Guidelines (Nabors et al., 2020).

All clinical and outcome data were collected through medical record reviews and telephone follow-ups conducted by two radiation oncologists. These data were subsequently confirmed by a senior radiation oncology specialist. Before initiating RT, baseline data were recorded, including age, sex, smoking history, pathological type, number and distribution of BM, and presence edema. Complete blood counts were performed to calculate systemic inflammatory and immune-related indices based on blood cell ratios, such as platelet-to-lymphocyte ratio (PLR), neutrophil-to-lymphocyte ratio (NLR), monocyte-to-lymphocyte ratio (MLR), systemic immune-inflammation index (SII), and systemic inflammation response index (SIRI). The calculation formulas are: PLR = platelet count/lymphocyte count; NLR = neutrophil count/lymphocyte count; MLR = monocyte count/lymphocyte count; SII = (neutrophil count × platelet count)/lymphocyte count; SIRI = (neutrophil count × monocyte count)/lymphocyte count.

All patients had their brain MRI reviewed two to three months after radiotherapy and efficacy was assessed using the RANO-BM criteria (Lin et al., 2015). Patients with complete or partial response were classified as responsive (CP group), while those with stable or progressive disease were categorized as non-responsive (SP group).

### Collection of fecal samples and 16S rRNA gene sequencing

Fecal samples were collected from the middle portion of patients’ stools in sterile plastic containers during the morning three days prior to the initiation of RT, and stored at -80°C within one hour. The gut microbiome was analyzed through 16S rRNA gene sequencing, with all procedures strictly following relevant guidelines.

Microbial DNA was extracted with the QIAamp DNA Stool Mini Kit (Qiagen, Hilden, Germany) and amplified via PCR on an ABI 2720 thermal cycler (Thermo Fisher Scientific, USA). DNA quantification was performed using a Multiskan™ GO spectrophotometer (Thermo Fisher Scientific, USA), and the V3-V4 regions of the 16S rRNA gene were amplified with Illumina adapter primers: forward (5′-CCTACGGGNGGCWGCAG-3′) and reverse (5′-GACTACHVGGGTATCTAATCC-3′). PCR products were purified with Agencourt AMPure XP beads (Beckman Coulter, USA), amplification was subsequently performed using TopTaq DNA Polymerase (Transgen, China). DNA purity and concentration were assessed using a NanoDrop 2000 spectrophotometer (Thermo Fisher Scientific, USA). Sequencing was conducted using paired-end (PE 250 bp) technology on the Illumina HiSeq 2500 platform by Treatgut Biotechnology Co., Ltd. (San Diego, CA, USA).

Paired-end reads were assembled with FLASH (Magoč and Salzberg, 2011), and primers and low-quality reads were removed using Cutadapt (Martin, 2011). Sequences were then clustered into OTUs at 97% similarity using Usearch (v10.0.240) (Edgar, 2013). Representative OTU sequences were classified using the RDP classifier (Wang et al., 2007) against the SILVA132 database (Quast et al., 2013), and aggregated at various taxonomic levels.

### Bioinformatics analysis of the gut microbiome

Dilution curve analysis (**Supplementary Figure 1A**) showed that the sequencing data had reached a plateau, ensuring sufficient sequencing depth to capture sample diversity. Additionally, Good’s Coverage index (**Supplementary Material**) was calculated to assess sequencing completeness, confirming that the majority of the microbial diversity was captured. Alpha diversity analysis evaluated species richness and evenness within samples using metrics such as observed OTUs, Chao1, ACE, Shannon, Simpson, and Pielou’s evenness index. Differences between the CP and SP groups were analyzed using the Wilcoxon rank-sum test. Beta diversity was assessed using Bray- Curtis distance-based principal coordinates analysis (PCoA) to visualize variations in community structure among groups. Inter-group differences were assessed using analysis of similarity (ANOSIM) and permutational multivariate analysis of variance (PERMANOVA) with 999 permutations. Linear discriminant analysis effect size (LEfSe) was used to identify microbial taxa with significant abundance differences between the CP and SP groups. A linear discriminant analysis (LDA) threshold of 2.0 was applied to assess discriminative ability (Paulson et al., 2013). Microbial functional prediction was performed was performed using PICRUSt software (Langille et al., 2013), and potential metabolic pathways and biological functions were annotated by integrating the Kyoto Encyclopedia of Genes and Genomes (KEGG) database (Kanehisa et al., 2016) and the Gene Ontology (GO) database (Ashburner et al., 2000).

### Predictive factor selection and model construction

We applied the least absolute shrinkage and selection operator (LASSO) regression model for variable selection. Ten-fold cross-validation was used to determine the optimal regularization parameter λ. Microbial features and clinical variables with non-zero coefficients were then identified. The chosen variables were analyzed to univariate logistic regression analysis to determine odds ratios (OR) and 95% confidence intervals (CI), assessing their relationship with RT response. Significant variables were included in a multivariate logistic regression model to control for confounders and identify independent predictors. We used linear regression outcomes from multivariate logistic regression analysis as microbial scores, integrating them with clinical factors to develop a nomogram model for personalized prediction of patient efficacy. The model’s predictive performance was evaluated with receiver operating characteristic (ROC) curves, and its clinical utility was shown using decision curve analysis (DCA).

### Statistical analysis

Normally distributed continuous variables were expressed as means with standard deviations (Mean ± SD) and compared using independent samples t-tests. Skewed continuous variables were expressed as medians with interquartile ranges (Median [IQR]) and analyzed using the Wilcoxon rank-sum test. Categorical variables were presented as frequencies and percentages (n, %) and compared using Chi-squared tests or Chi-squared tests, with or without Yates’ correction. Analyses were conducted using R software version 4.3.1. The primary R packages used were: tableone (v0.13.2) for baseline data statistical analysis, glmnet (v4.1-8) for LASSO regression, pROC (v1.18.5) for ROC analysis, rms (v6.4.0) for nomogram analysis, rmda (v1.6) for DCA, microeco (v1.10.0) for gut microbiome alpha and beta diversity and LEfSe analysis, and ggplot2 (v3.5.1) for data visualization. P-values < 0.05 were considered statistically significant. For multiple hypothesis testing, the Benjamin-Hochberg procedure was applied to adjust the P-values.

## Results

### Clinical baseline characteristics of patients

After applying the inclusion and exclusion criteria, 5 patients were excluded due to missing efficacy evaluation data, and 2 were excluded for not completing the RT plan, leaving 55 patients in the final cohort. **Table 1** presents clinical data statistics, showing a mean patient age of 63.18 years (range: 44.0-84.0). The cohort comprised of 38 males and 17 females, with 28 patients in the CP group and 27 in the SP group. The CP and SP groups exhibited no significant differences in age, sex, smoking history, pathological type, number and distribution of BM, or presence of edema (P> 0.05).C-reactive protein (C-RP) levels differed significantly between the groups (P= 0.001), with the SP group exhibiting a median of 13.5 mg/L, notably higher than the CP group’s median of 3.3 mg/L.

**Table 1:** Clinical baseline characteristics of CP and SP groups.

Characteristics	CP group (n=28)	SP group (n=27)	*P*
Age, y			0.349
Mean ± SD	62.00 (9.65)	64.41 (9.24)	
Sex, n (%)			0.281
Male	17 (60.7)	21 (77.8)	
Female	11 (39.3)	6 (22.2)	
Body mass index (kg/m^2^)			0.145
Mean ± SD	22.02 (4.39)	23.55 (3.17)	
Smoking history, n (%)			>0.999
Yes	11 (39.3)	11 (40.7)	
NO	17 (60.7)	16 (59.3)	
ECOG PS, n (%)			0.895
0-1	15 (53.6)	13 (48.1)	
2-4	13 (46.4)	14 (51.9)	
Pathology, n (%)			0.075
Adenocarcinoma	17 (60.7)	21 (77.8)	
Squamous cell carcinoma	1 (3.6)	3 (11.1)	
Small cell carcinoma	10 (35.7)	3 (11.1)	
Number of brain metastases, n (%)			0.162
<4	17 (60.7)	22 (81.5)	
≥4	11 (39.3)	5 (18.5)	
Frontal lobe metastasis, n (%)			0.700
Yes	18 (64.3)	15 (55.6)	
NO	10 (35.7)	12 (44.4)	
Parietal lobe metastasis, n (%)			0.869
Yes	12 (42.9)	10 (37.0)	
NO	16 (57.1)	17 (63.0)	
Temporal lobe metastasis, n (%)			0.074
Yes	16 (57.1)	8 (29.6)	
NO	12 (42.9)	19 (70.4)	
Occipital lobe metastasis, n (%)			>0.999
Yes	16 (57.1)	15 (55.6)	
NO	12 (42.9)	12 (44.4)	
Cerebellar hemisphere metastasis, n (%)			>0.999
Yes	12 (42.9)	11 (40.7)	
NO	16 (57.1)	16 (59.3)	
Edema around metastatic lesions, n (%)			>0.999
Yes	12 (42.9)	11 (40.7)	
NO	16 (57.1)	16 (59.3)	
Radiotherapy techniques, n (%)			0.640
FSRT	10 (35.7)	13 (48.1)	
WBRT	12 (42.9)	9 (33.3)	
WBRT+SIB	6 (21.4)	5 (18.1)	
BED (α/β=10)			0.394
Median (IQR)	48.00 (44.91, 56.00)	52.20 (45.94, 57.25)	
White blood cell count (10^9^/L)			0.106
Median (IQR)	5.47 (4.46, 7.40)	5.92 (5.16, 10.01)	
Absolute neutrophil count (10^9^/L)			0.165
Median (IQR)	4.02 (2.98, 5.39)	4.59 (3.30, 8.30)	
Absolute monocyte count (10^9^/L)			0.141
Mean ± SD	0.44 (0.18)	0.52 (0.18)	
Absolute lymphocyte count (10^9^/L)			0.973
Median (IQR)	1.01 (0.78,1.38)	1.07 (0.72,1.50)	
Platelet Count (10^9^/L)			0.743
Mean ± SD	192.64 (58.98)	198.74 (77.15)	
C-RP (mg/L)			**0.001**
Median (IQR)	3.30 (1.56, 7.12)	13.50 (8.36, 35.65)	
NLR			0.143
Median (IQR)	4.00 (2.36, 5.46)	5.10 (2.91, 8.28)	
MLR			0.136
Median (IQR)	0.38 (0.31, 0.48)	0.47 (0.37, 0.61)	
PLR			0.775
Median (IQR)	156.67 (114.01, 267.83)	177.42 (139.07, 254.28)	
SII			0.195
Median (IQR)	683.18 (426.88, 1028.34)	788.76 (585.96, 1782.86)	
SIRI			0.074
Median (IQR)	1.62 (0.91, 2.64)	2.10 (1.31, 5.14)	

CP group, radiotherapy responders; SP group, radiotherapy non-responders; ECOG PS, Eastern Cooperative Oncology Group performance status; SRT, stereotactic radiation therapy; WBRT, whole-brain radiotherapy; SIB, simultaneous integrated boost; BED, biologically effective dose; C-RP, c-reactive protein; NLR, neutrophil to lymphocyte ratio; MLR, monocyte to lymphocyte ratio; PLR, platelet to lymphocyte ratio; SII, systemic immune inflammation index; SIRI, systemic inflammation response index. Bolded values indicate P-values < 0.05.

### Alpha and beta diversity analysis

**Figure 2:**
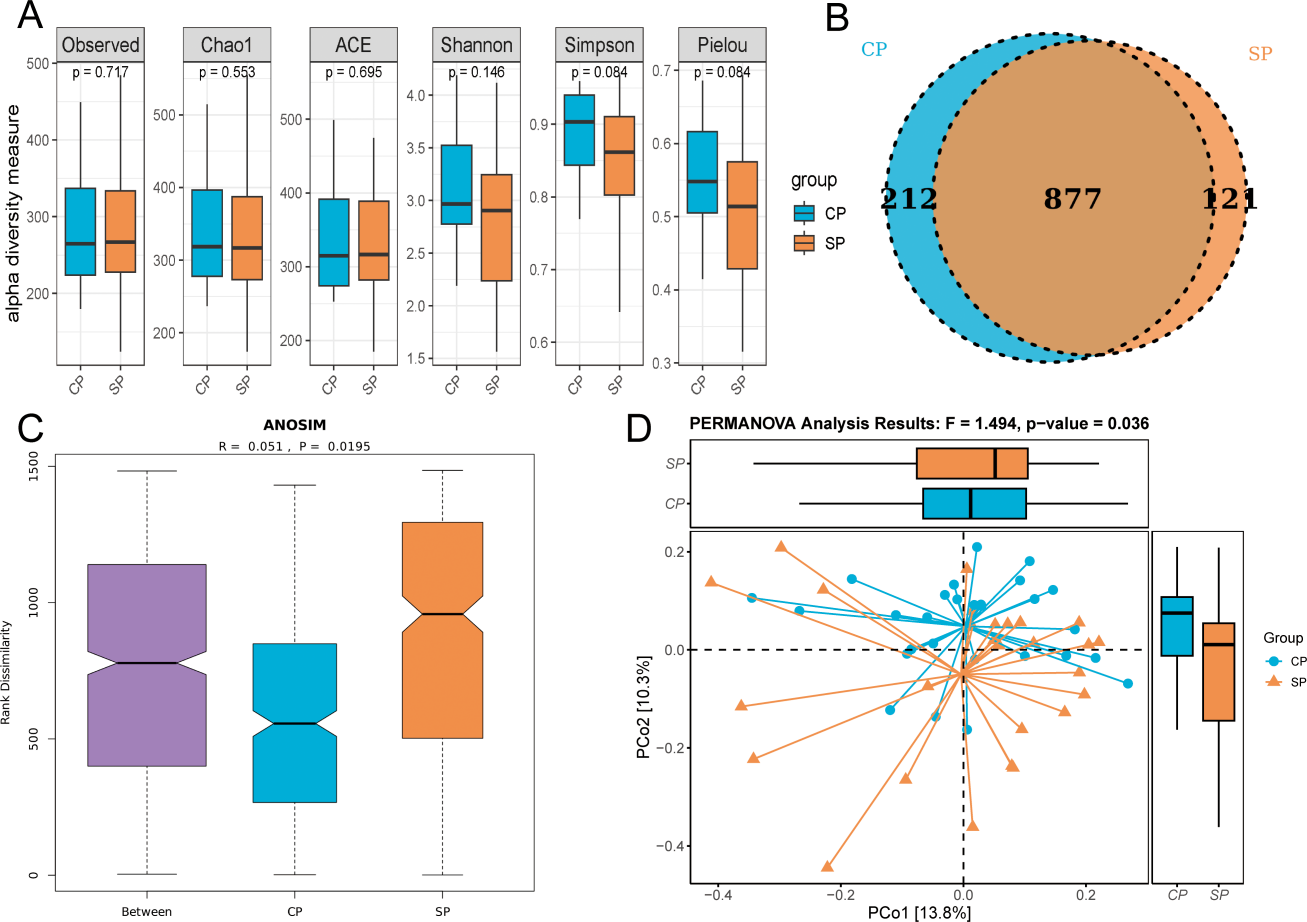
Alpha and beta diversity analysis. CP group, radiotherapy responders; SP group, radiotherapy non-responders. **(A)** Multiple indices showed no significant difference in α-diversity between the CP and SP groups. **(B)** Venn diagram illustrates the shared and unique OTUs between the two groups. **(C)** ANOSIM revealed a significant structural difference in microbial communities between the CP and SP groups (p = 0.0195). **(D)** PCoA plot visualized the distribution of microbial community structure, PERMANOVA confirmed significant differences in microbial composition between the CP and SP group (p = 0.036).

We assessed the alpha diversity of the gut microbiome in the CP and SP groups using Observed, Chao1, ACE, Shannon, Simpson, and Pielou’s evenness indices (**Figure 2A**). The findings revealed no significant differences between the groups across these indices (all P> 0.05), suggesting comparable richness, evenness, and diversity. A Venn diagram (**Figure 2B**) illustrates the common and distinct OTUs between the two groups. The CP group shared 877 OTUs and had 212 unique OTUs, whereas the SP group had 121 unique OTUs. In the beta diversity analysis, ANOSIM revealed a significant difference in microbial community distribution between the CP and SP groups (P= 0.0195) (**Figure 2C**). PCoA using the Bray-Curtis distance matrix (**Figure 2D**) supported this finding, with PCoA1 and PCoA2 accounting for 13.8% and 10.3% of the variance, respectively (PERMANOVA, F=1.49, P= 0.036).

### LEfSe analysis and functional annotation

**Figure 3:**
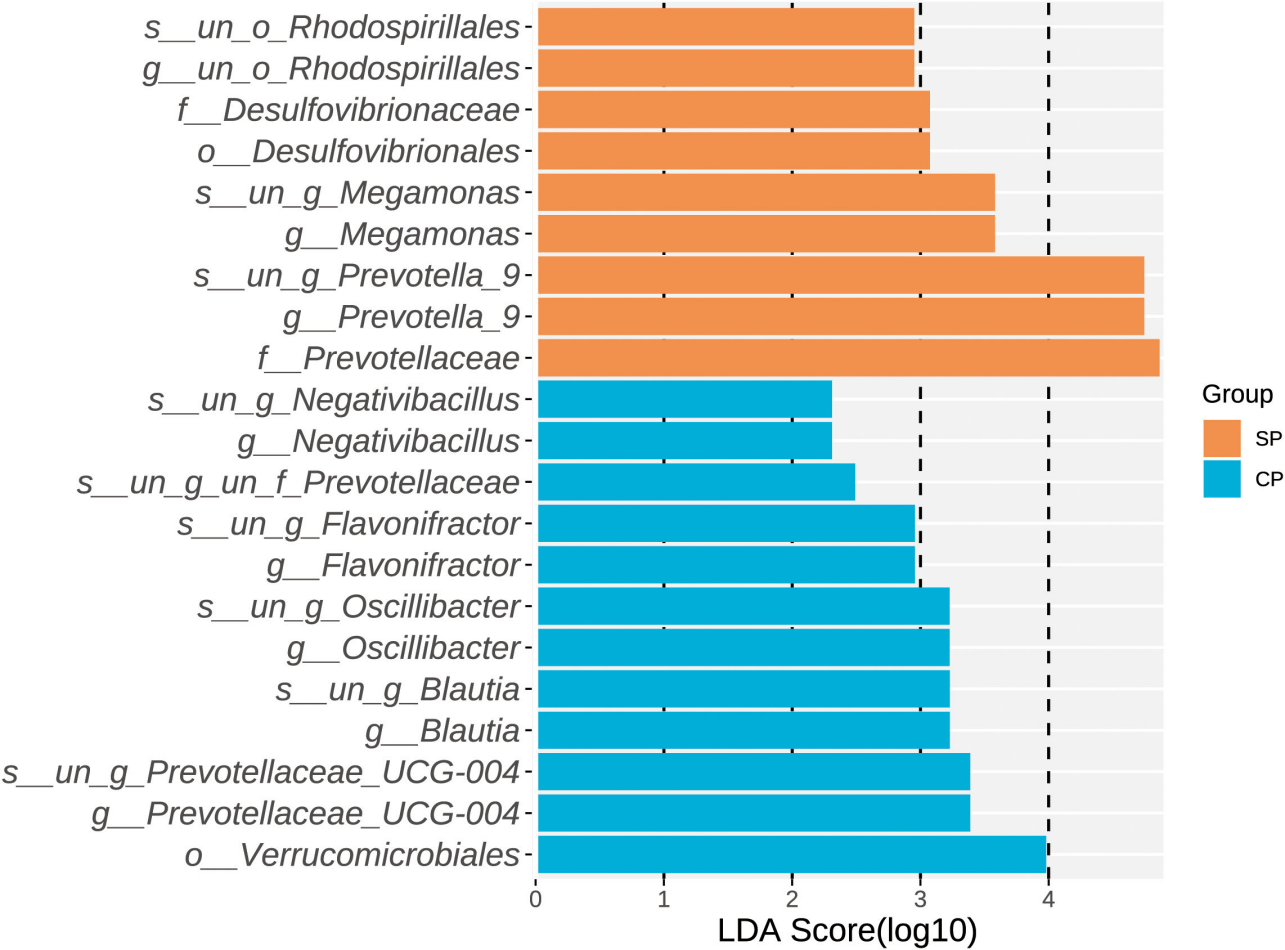
LDA scores of differential microbial taxa. LEfSe analysis identified 21 microbial taxa with significant differences between the CP and SP groups, all with LDA scores greater than 2. Twelve taxa were enriched in the CP group, while nine taxa were enriched in the SP group. CP group, radiotherapy responders; SP group, radiotherapy non-responders; LEfSe, linear discriminant analysis effect size; LDA, linear discriminant analysis.

Through LEfSe analysis (**Figure 3** for LDA scores, **Supplementary Table 1**), we identified a total of 21 microbial taxa with significant differences between the CP and SP groups. Among others, twelve characteristic microbes were significantly enriched in the CP group, including *Verrucomicrobiales* (P= 0.039) at the order level, *Prevotellaceae UCG-004* (P= 0.013) at the genus level, *Blautia* (P= 0.035), *Oscillibacter* (P= 0.029), *Flavonifractor* (P= 0.002), and *Negativibacillus* (P= 0.040). Conversely, nine characteristic microbes were significantly enriched in the SP group, including *Desulfovibrionaceae* (P= 0.038) at the family level, *Prevotellaceae* (P= 0.023), *Prevotella_9* at the genus level (P= 0.027), *Megamonas* (P= 0.029), and the unclassified order *Rhodospirillales* (P= 0.035).

KEGG database annotation analysis (**Supplementary Figure 2A**) revealed that, relative to the CP group, the SP group was significantly enriched in metabolic pathways related to transcription-related proteins, meiosis–yeast, cellular antigens, amyotrophic lateral sclerosis, and synthesis and degradation of ketone bodies. Additionally, GO database analysis (**Supplementary Figure 2B**) identified 20 different pathways to elucidate potential interaction patterns. Correlation analysis results (**Supplementary Figures 2C, D**) indicated that the *Desulfovibrionaceae* family was strongly correlated with multiple metabolic pathways and functional categories.

### Construction of efficacy prediction model based on microbial features

**Figure 4:**
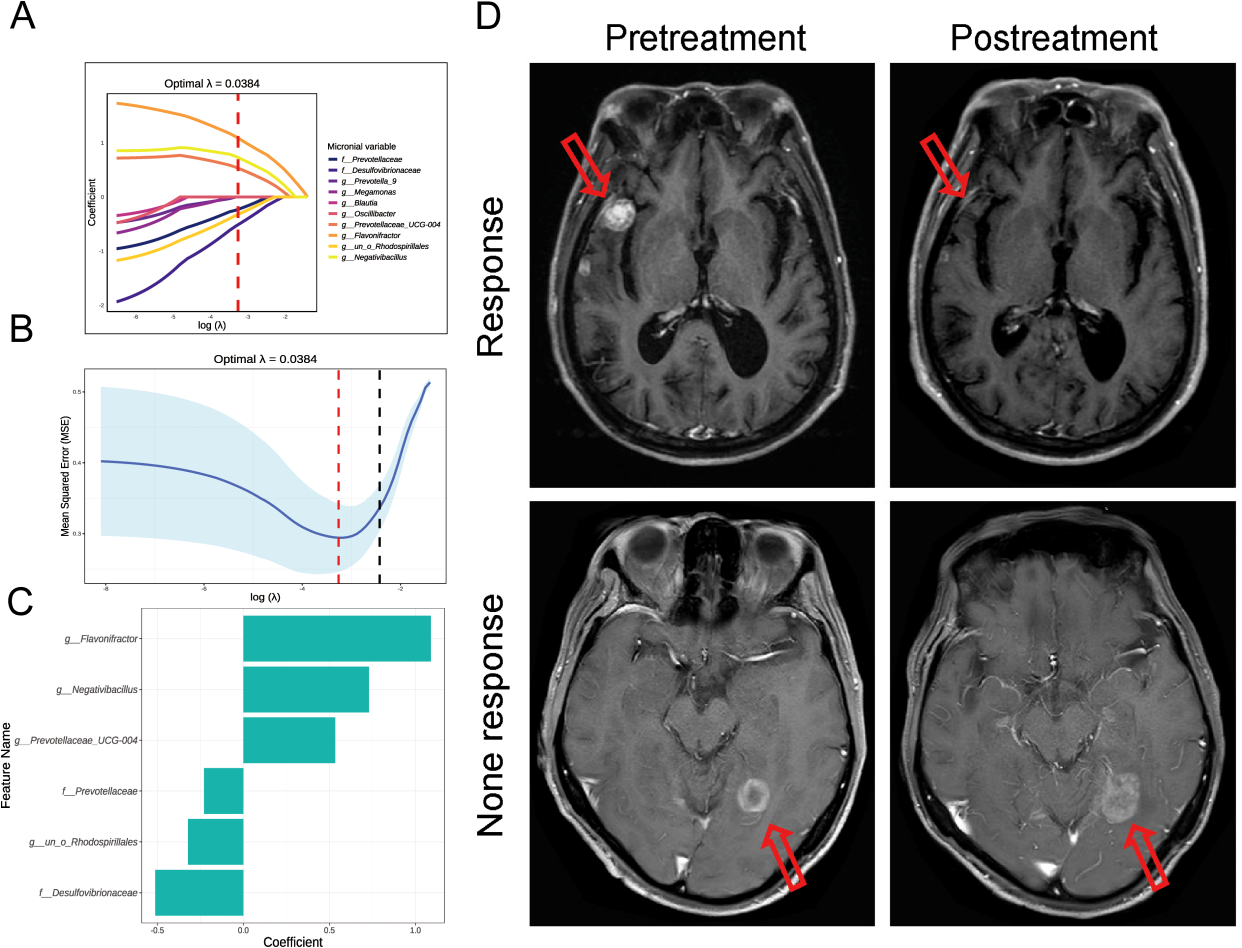
Construction of the LASSO model (microbial factors) and brain MRI scans of representative clinical cases. **(A)** Coefficient path plot for the 10 microbial features included. **(B)** Results of 10-fold cross-validation. **(C)** Six key microbial features selected by the LASSO model. **(D)** Upper panel: Patient 1 had a microbial score of 0.317, CRP of 10.75, and SIRI of 1.363. Using these values into the nomogram model formula, the linear predictor was -0.4306, which corresponds to a risk probability of 0.394 for no response. Post-treatment MRI showed near-complete resolution of the irradiated lesion, and the clinical outcome was consistent with the model’s prediction. Lower panel: Patient 2 had a microbial score of 1.339, CRP of 13.5, and SIRI of 5.005. Using the nomogram formula, the linear predictor was 2.0427, corresponding to a risk probability of 0.885 for no response. Follow-up MRI after radiotherapy indicated disease progression, and the clinical outcome matched the model’s prediction.

We selected the abundance of 10 microbes as candidate features. These features were derived from the LEfSe analysis and include significantly enriched microbial taxa at the family and genus levels in both the CP and SP groups. In the 10-fold cross-validation of the LASSO model, the optimal lambda parameter, yielding the minimum mean squared error (MSE) of 0.0384, resulted in six features with non-zero coefficients (**Figures 4A–C**). Univariate logistic regression analysis revealed that the abundance of five microbial features was significantly associated with treatment response, including *g_Flavonifractor* (OR= 3.667, P= 0.001), *g_Negativibacillus* (OR=2.131, P=0.015), *f_ Prevotellaceae* (OR= 0.486, P= 0.019), *f_ Desulfovibrionaceae* (OR= 0.471, P= 0.020), and *g_Prevotellaceae_UCG-004* (OR= 2.594, P= 0.039). Further multivariate logistic regression analysis indicated that *g_Flavonifractor* (OR= 6.680, P= 0.004) and *g_Negativibacillus* (OR= 3.862, P=0.014) were independent predictors of RT response, both of which were significantly enriched in the CP group. The same analytical approach was applied to clinical factors. The LASSO model selected eight features with non-zero coefficients from 20 clinical variables, with the optimal lambda parameter at 0.0548 (**Supplementary Figures 3A–C**). By combining univariate and multivariate logistic regression analyses, we identified C-RP (OR=1.054, P=0.017) and SIRI (OR=1.367, P=0.043) as independent clinical variables distinguishing the CP and SP groups. These variables were closely associated with the SP group’s treatment response. The comprehensive analysis results are summarized in **Table 2**.

**Table 2:** Results of LASSO, univariate, and multivariate analyses of potential variables associated with radiotherapy response in lung cancer patients with brain metastases.

Variable	LASSO coefficient	Univariate analysis		Multivariate analysis	
		OR (95% CI)	*P*	OR (95% CI)	*P*
*g_ Flavonifractor*	1.08851545	3.667 (1.657-8.119)	**0.001**	6.680 (3.320-13.528)	**0.004**
*g_ Negativibacillus*	0.7302148	2.131 (1.156-3.925)	**0.015**	3.862 (1.950-7.658)	**0.014**
*f_ Prevotellaceae*	-0.2282743	0.486 (0.265-0.89)	**0.019**	0.723 (0.295-1.763)	0.529
*f_ Desulfovibrionaceae*	-0.51178488	0.471 (0.250-0.89)	**0.020**	0.352 (0.126-0.976)	0.115
*g_ Prevotellaceae_UCG-004*	0.53343886	2.594 (1.051-6.399)	**0.039**	3.347 (1.459-7.695)	0.081
*g_ un_ o_ Rhodospirillales*	-0.32158835	0.533 (0.277-1.024)	0.059		
Pathology (Small cell carcinoma)	9.65E-01	2.429 (0.231-25.511)	0.460		
Sex (Female)	-6.96E-02	0.442 (0.135-1.441)	0.175		
Absolute monocyte count	-1.26E+00	0.1 (0.005-2.193)	0.144		
CRP	-2.73E-02	0.95 (0.91-0.992)	**0.021**	1.054 (1.009-1.100)	**0.017**
SIRI	-4.99E-02	0.761 (0.58-0.999)	**0.049**	1.367 (1.009, 1.854)	**0.043**
PLR	8.19E-05	1 (0.995-1.006)	0.863		
NLR	-2.67E-02	0.847 (0.712-1.008)	0.062		
Number of brain metastases (≥4)	8.25E-01	2.847 (0.83-9.761)	0.096		

CI, confidence; OR, odds ratio; CRP, c-reactive protein; SIRI, systemic inflammation response index; PLR, platelet to lymphocyte ratio; NLR, neutrophil to lymphocyte ratio.

Bolded values indicate P-values < 0.05.

**Figure 5:**
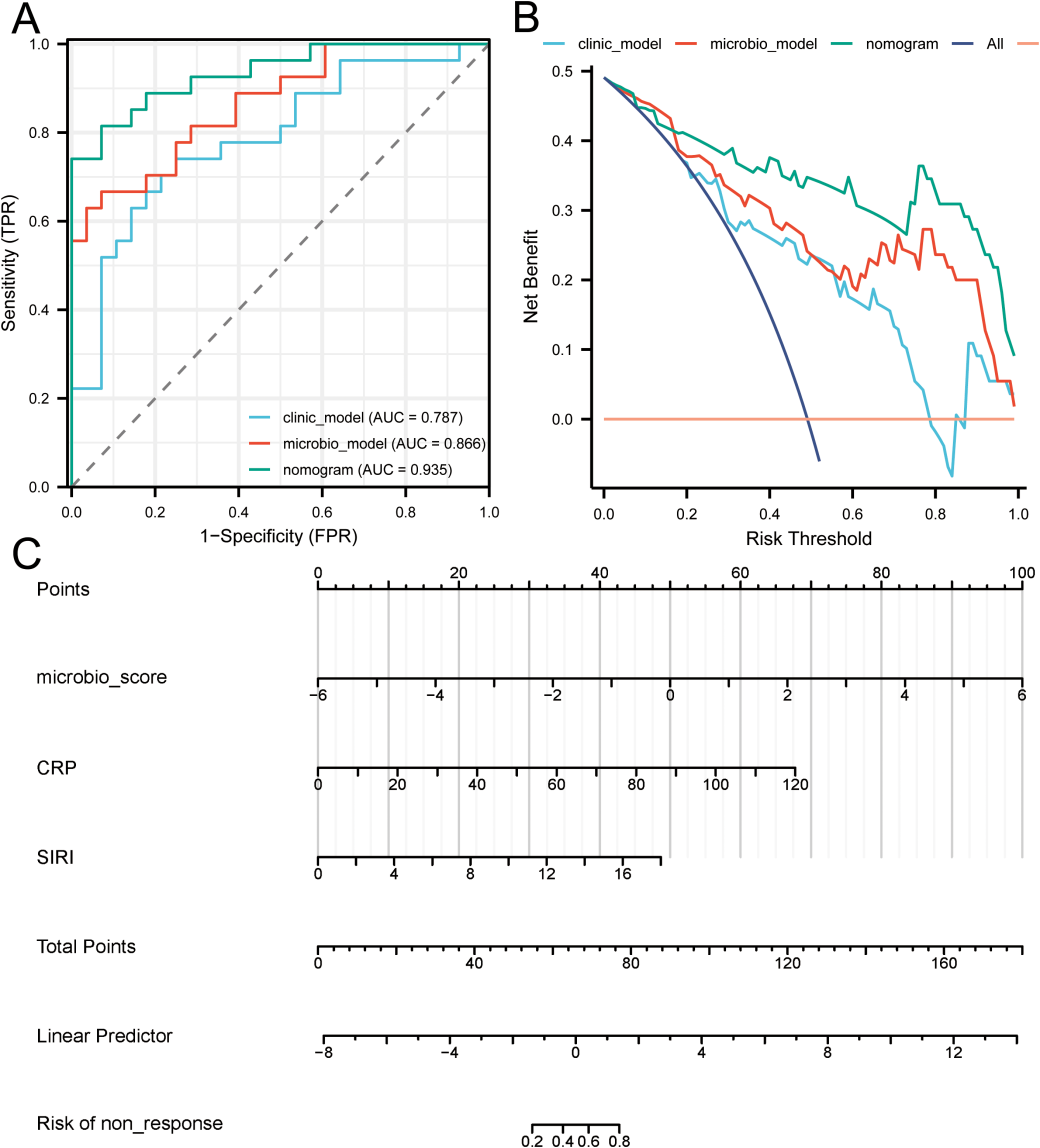
Evaluation of predictive model performance. **(A)** Presents the ROC curves for the microbial model, clinical model, and combined nomogram model, indicating their respective AUC values. **(B)** Provides a DCA comparing the net benefits of the microbial model, clinical model, and combined nomogram model across various risk thresholds. **(C)** Depicts the nomogram integrating microbial scores and clinical factors, illustrating their combined effect on risk prediction.

The ROC curves (**Figure 5A**) for the microbial model, clinical model, and combined nomogram model demonstrated superior predictive performance for both the nomogram and microbial models, with area under the curve (AUC) values of 0.935 and 0.866, respectively, compared to the clinical model’s AUC of 0.787. Further DCA (**Figure 5B**) revealed that, at various risk thresholds, both the microbial and nomogram models provided net benefits for patients, whereas the clinical model did not offer net benefits at some thresholds. The nomogram model (**Figure 5C**) illustrates the relationship between the microbial score and clinical factors in the combined model. Specifically, the microbial score was calculated as: -0.07872 + (-1.87004 × *g_Flavonifractor*) + (-1.49926 × *g_Negativibacillus*). Using this scoring system, a cutoff value of 0.72276 was established to differentiate patients who responded to RT for BM (Score < 0.72276) from those who did not (Score ≥ 0.72276). The mathematical formula for the nomogram model is: -1.97072 + microbial score × 1.03512 + CRP × 0.07014 + SIRI × 0.33582. Finally, in **Figure 4D**, we present pre- and post-treatment brain MRI images of two representative patients, clearly demonstrating the clinical applicability and effectiveness of the nomogram model.

## Discussion

Radiotherapeutic responses in LC-BM patients are highly heterogeneous, and the underlying mechanisms remain unclear. This study provides a preliminary analysis of the gut microbiome in RT responders (CP group) and non-responders (SP group). The study found no significant differences in microbial richness, evenness, or diversity between the two groups. However, distinct differences were observed in microbial community composition. The CP group was enriched with genera such as *Blautia* and *Oscillibacter*, while the SP group showed significant enrichment of genera like *Megamonas*. Metabolic pathways associated with treatment response included ketone body metabolism and pathways related to amyotrophic lateral sclerosis. Furthermore, a predictive model incorporating gut microbial features (*g_Flavonifractor* and *g_Negativibacillus*) and clinical variables (C-RP and SIRI) demonstrated high predictive accuracy (AUC = 0.935). These findings suggest that the model could potentially serve as a valuable tool for the early clinical identification of patients who are likely to exhibit poor responses to RT.

As previously mentioned, the microbiome residing in the host’s intestinal epithelium is essential for modulating the efficacy of antitumor therapies. The gut microbiota significantly influences the outcomes of chemotherapy, immunotherapy, and RT by regulating the host immune system and producing metabolic byproducts. In murine models treated with cyclophosphamide, a chemotherapeutic agent, the ability to suppress tumor growth is markedly reduced in germ-free or antibiotic-treated mice due to the absence of key subsets of helper T lymphocytes (Th1 and Th17) (Viaud et al., 2013). Supplementation with *Enterococcus hirae* and *Barnesiella intestinihominis* induces the generation of Th1, Th17, and tumor-specific CD4^+^ and CD8^+^ T cells, thereby restoring the antitumor effects of cyclophosphamide (Daillère et al., 2016). In the realm of immunotherapy, Vétizou et al. (2015) demonstrated that oral administration of *Mycobacterium fragilis* in combination with *Bacteroides thetaiotaomicron* or *Burkholderia cepacia* activates Th1 responses in lymph nodes. This promotes the maturation of dendritic cells within tumors, thereby enhancing the antitumor efficacy of CTLA-4 blockade. Radiotherapy exerts its effects by locally destroying the DNA molecules of cancer cells through high-energy radiation, which leads to impaired cell division and proliferation, and ultimately inducing cell death. Research on the influence of the gut microbiota on RT is relatively limited and can be categorized into effects on the digestive and non-digestive systems. In the digestive system, Dong et al. (2024) reported that butyrate derived from *Roseburia intestinalis* enhances the sensitivity of colorectal cancer to RT by activating the OR51E1/RALB axis and promoting autophagy. However, the specific mechanisms by which the gut microbiota regulates the efficacy of RT in non-digestive systems remain largely unknown, with existing studies providing only preliminary insights. Uribe-Herranz et al. (2020) demonstrated that Gram-positive gut bacteria can modulate antigen presentation by dendritic cells, thereby enhancing the local and distal effects of RT in cervical and lung cancers. Shiao et al. (2021) found that interactions between symbiotic bacteria and fungal communities within the gut microbiota jointly shape the tumor microenvironment in breast cancer. In murine models, depletion of gut symbiotic bacteria leads to fungal overgrowth, suppressing immune responses by modulating macrophage and T cell functions, thereby reducing the efficacy of RT.

Although direct evidence linking the gut microbiota to the RT of LC-BM is currently lacking, the microbiota-gut-brain axis (MGBA) may help explain our observed results (Mehrian-Shai et al., 2019). The MGBA is established through the circulatory, immune, and nervous systems, mediating bidirectional communication between the gut microbiome and the brain (Loh et al., 2024). Short-chain fatty acids (SCFAs), primarily including acetate, butyrate, and propionate, are the main metabolic products generated by the fermentation of dietary fibers and resistant starches by gut microbiota in anaerobic environments (Zhang et al., 2023). SCFAs not only alleviate tissue inflammation and maintain gut barrier function but also traverse the blood-brain barrier via the MGBA, where they regulate the maturation and function of resident immune cells in the brain (Mann et al., 2024). For instance, oral supplementation of SCFAs can increase the number of M1-type microglia in the tumor microenvironment by activating glycolysis pathways (Zhou et al., 2024). Liu et al. (2024) found that polarized M1-type microglia synergize with RT to enhance the radiosensitivity of non-small cell lung cancer brain metastases. In our study, the CP group was uniquely enriched with bacterial families *Ruminococcaceae* and *Lachnospiraceae*, including genera such as *Oscillibacter* and *Blautia*, which have been identified as major producers of SCFAs (Holmberg et al., 2024; Zhao et al., 2024). In contrast, the SP group was uniquely enriched with the family *Desulfovibrionaceae* and the genus *Megamonas*, both of which have been reported to be associated with chronic intestinal inflammation (Balmant et al., 2023). For example, bacteria of the order Desulfovibrionales possess sulfate-reducing genes that convert sulfate to H_2_S, disrupting the intestinal barrier and producing endotoxins and inflammatory cytokines like IL-6 (Hu et al., 2022). With the formation of intestinal wall inflammation and increased permeability, cytokines such as IL-6 can enter the brain via the MGBA, inducing neuroinflammation and neuronal death (Kustrimovic et al., 2024). Studies have shown that cytokines like IL-6 are associated with radioresistance, potentially leading to reduced therapeutic efficacy in patients (Zhao et al., 2021). Furthermore, metabolic pathway analysis of the SP group (e.g., pathways related to amyotrophic lateral sclerosis) also suggests a high-inflammatory state within the brain of these patients (Zhang et al., 2021b). For example, TDP-43 (TAR DNA-binding protein 43) facilitates the release of mitochondrial DNA through the mitochondrial permeability transition pore, which activates the cGAS-STING signaling pathway and subsequently promotes the release of pro-inflammatory cytokines, such as IFN-β, IL-6, TNF, and IL-1β (Yu et al., 2020). This mechanism may play a critical role in radiotherapy resistance. Additionally, PPAR (peroxisome proliferator-activated receptor) activation, through the regulation of antioxidant, pro-proliferative, and anti-apoptotic pathways, may also contribute to the development of radiotherapy resistance (Zhang et al., 2024). Meanwhile, SOD1 (superoxide dismutase 1)’s role in inhibiting ROS (reactive oxygen species) accumulation, maintaining cellular antioxidant capacity, and regulating cell cycle responses may enhance cellular resistance to radiation (Gao et al., 2008). We believe these amyotrophic lateral sclerosis-related pathways offer new perspectives for studying radiotherapy resistance and merit further exploration. In the clinical data of SP group patients, inflammatory markers such as C-RP were significantly higher than those in the CP group, seemingly adding further evidence. In summary, we speculate that the better radiotherapeutic response in CP group patients may be linked to SCFA-producing microbiota enhancing RT’s antitumor effects by regulating microglia. In contrast, poorer efficacy in SP group patients may result from gut microbiota-induced intestinal inflammation, which allows inflammatory cytokines like IL-6 to enter the brain.

This study also identified the gut microbiota as potential biomarkers for predicting the radiotherapeutic response in LC-BM. By constructing predictive models based on microbial features and clinical variables, we found that the nomogram model and microbiota model achieved AUC of 0.935 and 0.866, respectively, outperforming the traditional clinical model (AUC = 0.787). DCA further validated that both models provided significant net benefits to patients across different risk thresholds, whereas the clinical model did not. Additionally, we discovered that inflammatory and immune-related indicators, C-RP and systemic SIRI, may serve as potential factors for predicting RT response, offering new references for the optimization of clinical treatment plans. Although previous studies have explored the predictive roles of inflammatory and immune-related indicators such as SII (Zhang et al., 2021a), PNI (Li et al., 2021), and PLR (Li et al., 2020) in RT for LC-BM, C-RP and SIRI demonstrated unique predictive potential in our study.

Indeed, there are several limitations in our study. The study’s small sample size and single-center design have been influenced by regional, ethnic, and dietary influences. Future studies should validate the generalizability of these findings through multi-center, large-scale cohort studies. Secondly, the study’s reliance solely on 16S rRNA gene sequencing, without incorporating metagenomics, metabolomics, or other multi-omics data, restricts a comprehensive understanding of the gut microbiota’s role in RT response mechanisms. The study’s cross-sectional design failed to capture dynamic changes in gut microbiota during RT. Longitudinal studies will elucidate the temporal relationship between microbiota changes and therapeutic outcomes. Finally, although we identified differential microbiota associated with RT response, we did not validate their causal relationships or specific mechanisms using animal models. In conclusion, this study offers a novel perspective on the gut microbiota’s role in the variability of RT response, despite its limitations.

## Conclusion

This study is the first to explore the variability in RT effectiveness among LC-BM patients by examining gut microbiota. Prior to RT, the composition and functionality of the gut microbiota were associated with treatment outcomes, highlighting its potential as a predictive biomarker for therapeutic efficacy. We suggest that the gut microbiota could affect RT effectiveness by modulating the brain’s immune microenvironment via the MGBA. Additionally, we identified C-RP and SIRI, two inflammatory immune-related indices, as potential predictors of RT response, a finding not previously reported. Our findings provide new insights into RT efficacy in LC-BM patients and pave the way for the clinical implementation of personalized treatment strategies.

## Data Availability

The data analyzed in this study is subject to the following licenses/restrictions: The Original data presented in this article are not publicly available due to patient privacy. Inquiries could be directed to the corresponding author with reasonable request. Requests to access these datasets should be directed to Xia Youyou, xia.youyou@njmu.edu.cn.
