# Supplementary material for: Gut microbiome is associated with radiotherapy response in lung cancer patients with brain metastases
Source: Front Cell Infect Microbiol. 2025 Mar 10;15:1562831. doi: 10.3389/fcimb.2025.1562831 (PMC11931136; doi:10.3389/fcimb.2025.1562831)
Supplement: Supplementary file 1 [file DataSheet1.zip › Supplementary figure & table.docx]

Supplementary Material


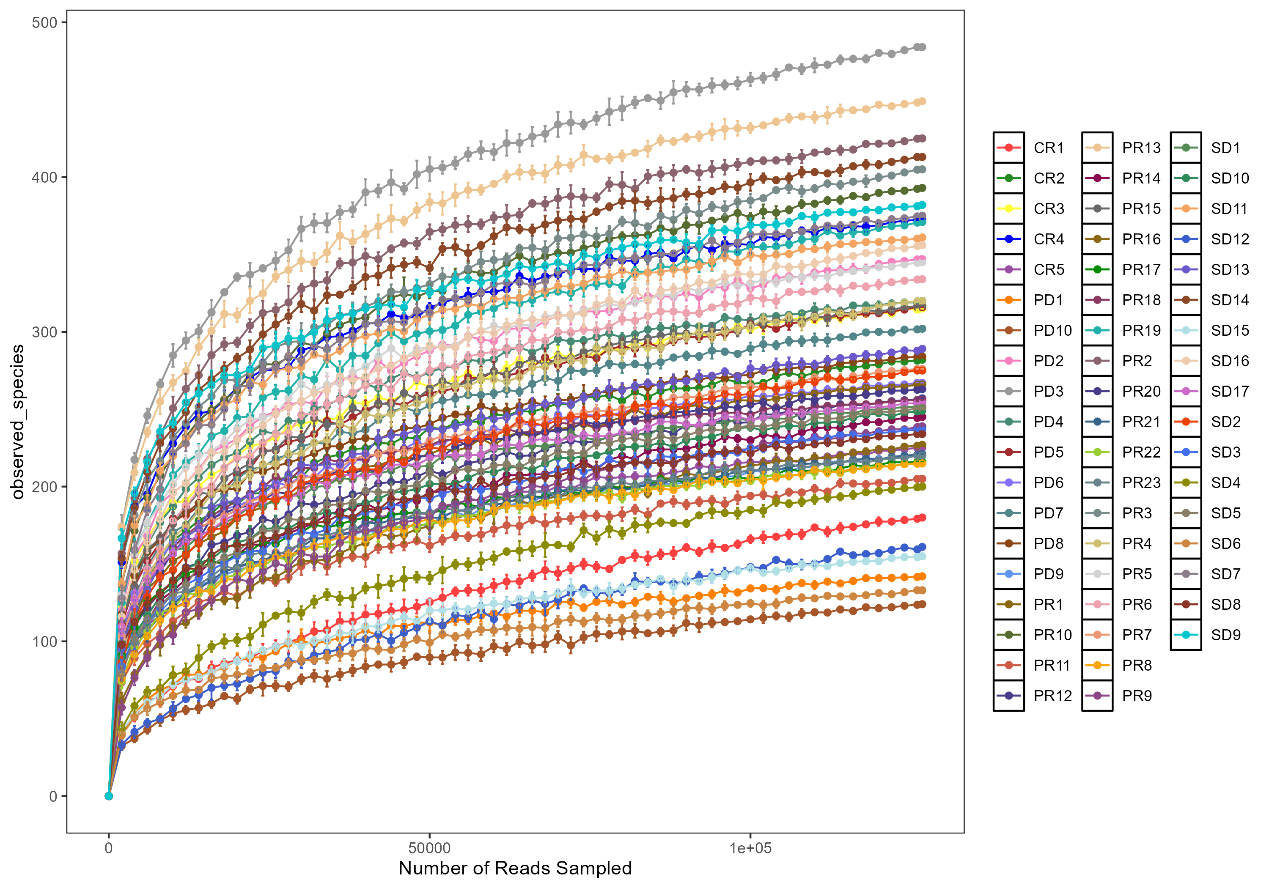


**Supplementary Figure 1**. Rarefaction Curves.

The rarefaction curves generated from the sequencing data were generally stable, indicating that the sequencing depth was sufficient to adequately capture the microbial diversity.


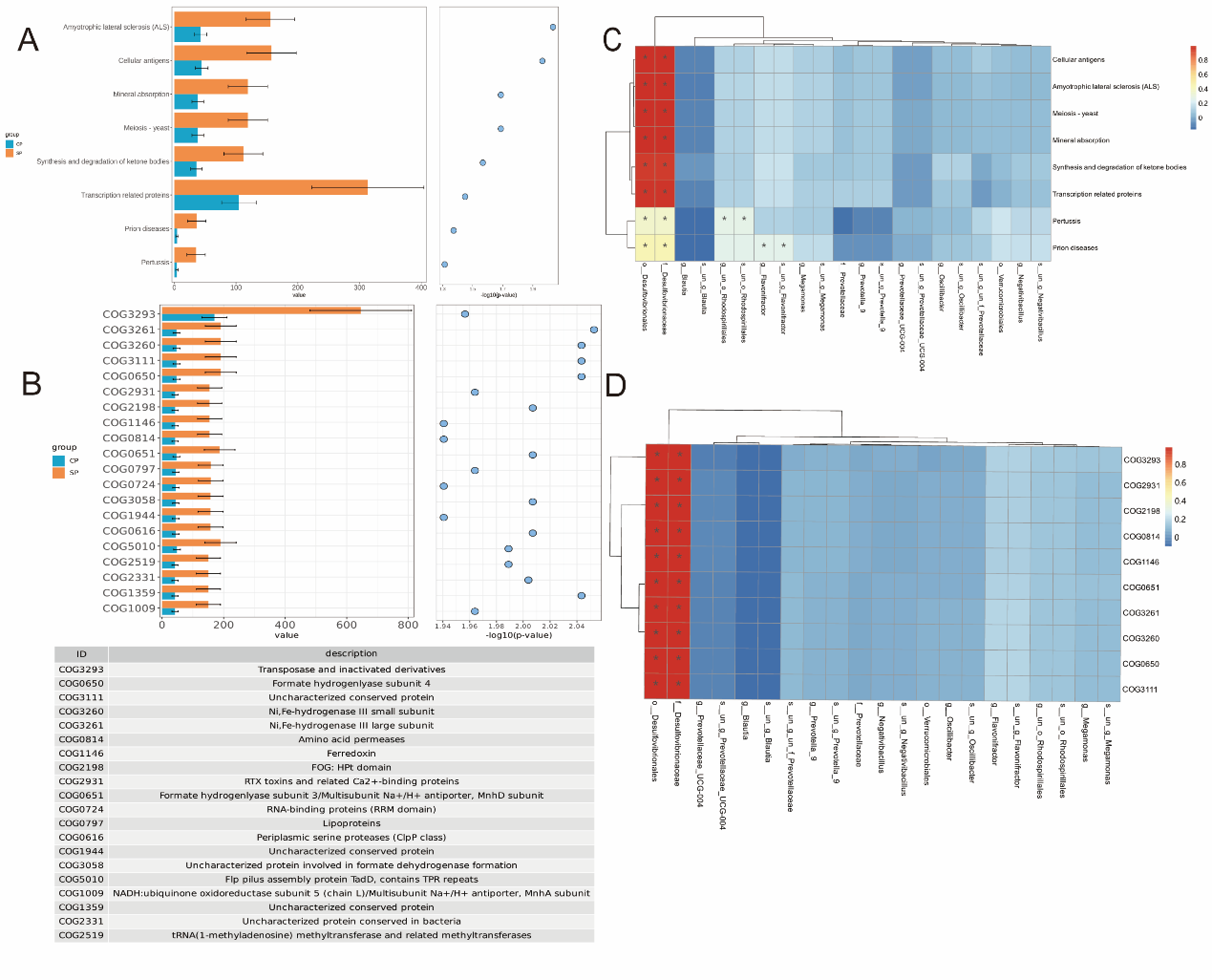


**Supplementary Figure 2**. Functional Annotation and Correlation Analysis.

KEGG (A) and Top 20 GO (B) annotation results of 21 differentially abundant microbes between CP and SP groups. Correlation analysis of 21 differentially abundant microbial taxa with 8 significant KEGG (C) metabolic pathways and the top 10 significant GO pathways (D). CP group= radiotherapy responders; SP group= radiotherapy non-responders.


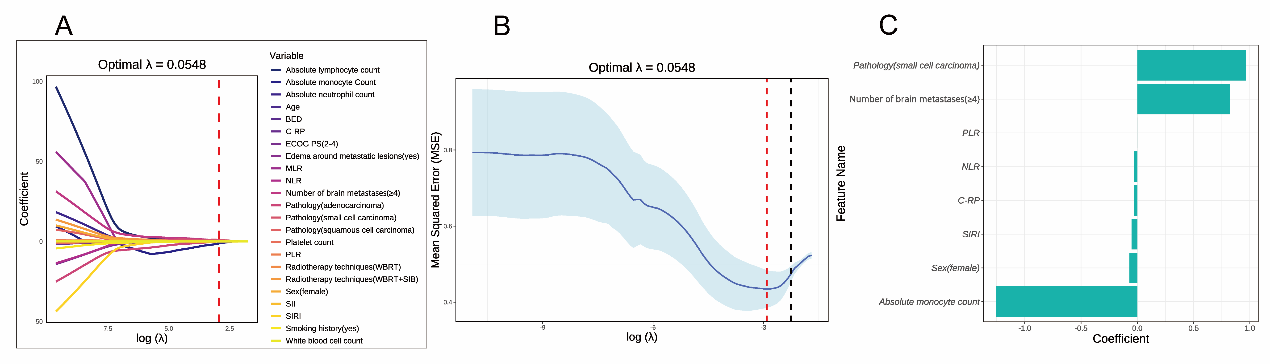


**Supplementary Figure 3.** Construction of the LASSO Model (clinical factors).

(A)Coefficient path plot for the 20 clinical features.

(B)Results of 10-fold cross-validation.

(C) Eight key clinical features selected by the LASSO model.

| **Supplementary Table1.** LEfSe Analysis Identifies Differentially Abundant Microbial Taxa Between CP and SP Groups. | | | | | | | |
| --- | --- | --- | --- | --- | --- | --- | --- |
| **Taxa** | **Group** | **N** | **Mean** | **SD** | **SE** | ***P*^*^** | **LDA**  **score** |
| *f_ Desulfovibrionaceae* | CP | 28 | 0.001343504 | 0.001641261 | 0.000310169 | 0.038 | 3.074 |
|  | SP | 27 | 0.003789187 | 0.005330381 | 0.001025832 |  |  |
| *f_ Prevotellaceae* | CP | 28 | 0.03660141 | 0.089826752 | 0.01697566 | 0.023 | 4.864 |
|  | SP | 27 | 0.17548142 | 0.276923917 | 0.053294033 |  |  |
| *g_ Blautia* | CP | 28 | 0.007958482 | 0.007827668 | 0.00147929 | 0.035 | 3.223 |
|  | SP | 27 | 0.004533001 | 0.004513155 | 0.000868557 |  |  |
| *g_ Flavonifractor* | CP | 28 | 0.00239994 | 0.004234013 | 0.000800153 | 0.002 | 2.957 |
|  | SP | 27 | 0.00073096 | 0.001668921 | 0.000321184 |  |  |
| *g_ Megamonas* | CP | 28 | 0.01026292 | 0.04852138 | 0.009169679 | 0.029 | 3.581 |
|  | SP | 27 | 0.005176316 | 0.009989759 | 0.00192253 |  |  |
| *g_ Negativibacillus* | CP | 28 | 0.000424546 | 0.00085684 | 0.000161928 | 0.040 | 2.311 |
|  | SP | 27 | 4.29461E-05 | 7.91329E-05 | 1.52291E-05 |  |  |
| *g_ Oscillibacter* | CP | 28 | 0.003758657 | 0.007794097 | 0.001472946 | 0.029 | 3.227 |
|  | SP | 27 | 0.000714015 | 0.000843058 | 0.000162247 |  |  |
| *g_ Prevotella_9* | CP | 28 | 0.021661438 | 0.086607737 | 0.016367324 | 0.027 | 4.745 |
|  | SP | 27 | 0.132516073 | 0.253213204 | 0.048730904 |  |  |
| *g_Prevotellaceae_UCG-004* | CP | 28 | 0.004207149 | 0.015464846 | 0.002922581 | 0.013 | 3.389 |
|  | SP | 27 | 2.3372E-06 | 6.85392E-06 | 1.31904E-06 |  |  |
| *g_ un_ o_ Rhodospirillales* | CP | 28 | 7.0429E-06 | 2.21386E-05 | 4.1838E-06 | 0.035 | 2.953 |
|  | SP | 27 | 0.001884076 | 0.008627828 | 0.001660426 |  |  |
| *o_ Desulfovibrionales* | CP | 28 | 0.001343504 | 0.001641261 | 0.000310169 | 0.036 | 3.074 |
|  | SP | 27 | 0.003789187 | 0.005330381 | 0.001025832 |  |  |
| *o_ Verrucomicrobiales* | CP | 28 | 0.021593263 | 0.07811907 | 0.014763117 | 0.039 | 3.980 |
|  | SP | 27 | 0.002150517 | 0.005885247 | 0.001132616 |  |  |
| *s_ un _ g_ Blautia* | CP | 28 | 0.007958482 | 0.007827668 | 0.00147929 | 0.035 | 3.229 |
|  | SP | 27 | 0.004533001 | 0.004513155 | 0.000868557 |  |  |
| *s_ un _ g_ Flavonifractor* | CP | 28 | 0.00239994 | 0.004234013 | 0.000800153 | 0.002 | 2.957 |
|  | SP | 27 | 0.00073096 | 0.001668921 | 0.000321184 |  |  |
| *s_ un_ g_ Megamonas* | CP | 28 | 0.01026292 | 0.04852138 | 0.009169679 | 0.029 | 3.581 |
|  | SP | 27 | 0.005176316 | 0.009989759 | 0.00192253 |  |  |
| *s_ un_ g _ Negativibacillus* | CP | 28 | 0.000424546 | 0.00085684 | 0.000161928 | 0.04 | 2.311 |
|  | SP | 27 | 4.29461E-05 | 7.91329E-05 | 1.52291E-05 |  |  |
| *s_ un_ g _ Oscillibacter* | CP | 28 | 0.003758657 | 0.007794097 | 0.001472946 | 0.029 | 3.227 |
|  | SP | 27 | 0.000714015 | 0.000843058 | 0.000162247 |  |  |
| *s_un_g_Prevotella_9* | CP | 28 | 0.021661438 | 0.086607737 | 0.016367324 | 0.027 | 4.745 |
|  | SP | 27 | 0.132516073 | 0.253213204 | 0.048730904 |  |  |
| *s_un_g_Prevotellaceae_UCG-004* | CP | 28 | 0.004207149 | 0.015464846 | 0.002922581 | 0.013 | 3.389 |
|  | SP | 27 | 2.3372E-06 | 6.85392E-06 | 1.31904E-06 |  |  |
| *s_ un_g_un_f_ Prevotellaceae* | CP | 28 | 0.00057949 | 0.003029405 | 0.000572504 | 0.023 | 2.491 |
|  | SP | 27 | 0 | 0 | 0 |  |  |
| *s_un_o_Rhodospirillales* | CP | 28 | 7.0429E-06 | 2.21386E-05 | 4.1838E-06 | 0.035 | 2.953 |
|  | SP | 27 | 0.001884076 | 0.008627828 | 0.001660426 |  |  |
| **Abbreviations:** LEfSe= linear discriminant analysis effect size; LDA= linear discriminant analysis; CP group= radiotherapy responders; SP group= radiotherapy non-responders; SD= standard deviation; SE= standard error. *= indicates FDR-corrected P-values. | | | | | | | |
